# Gibberellins negatively regulate the development of *Medicago truncatula* root system

**DOI:** 10.1038/s41598-019-38876-1

**Published:** 2019-02-20

**Authors:** Camille Fonouni-Farde, Ambre Miassod, Carole Laffont, Halima Morin, Abdelhafid Bendahmane, Anouck Diet, Florian Frugier

**Affiliations:** Institute of Plant Sciences Paris-Saclay (IPS2), CNRS, Univ Paris Diderot, INRA, Univ Paris Sud, Univ d’Evry, Université Paris-Saclay, Rue de Noetzlin, 91190 Gif-sur-Yvette France

## Abstract

The root system displays a remarkable plasticity that enables plants to adapt to changing environmental conditions. This plasticity is tightly linked to the activity of root apical meristems (RAMs) and to the formation of lateral roots, both controlled by related hormonal crosstalks. In *Arabidopsis thaliana*, gibberellins (GAs) were shown to positively control RAM growth and the formation of lateral roots. However, we showed in *Medicago truncatula* that GAs negatively regulate root growth and RAM size as well as the number of lateral roots depending at least on the MtDELLA1 protein. By using confocal microscopy and molecular analyses, we showed that GAs primarily regulate RAM size by affecting cortical cell expansion and additionally negatively regulate a subset of cytokinin-induced root expansin encoding genes. Moreover, GAs reduce the number of cortical cell layers, resulting in the formation of both shorter and thinner roots. These results suggest contrasting effects of GA regulations on the root system architecture depending on plant species.

## Introduction

Roots exhibit a remarkable developmental plasticity representing a key adaptive trait that enables plant adaptation to external conditions. Indeed, both primary root growth and root branching through lateral root formation are determined by the soil environment^1^. Root growth results from the activity of the root apical meristem (RAM), showing three main types of organization patterns in Eudicotyledonous plants, namely closed, basic-open and intermediate-open RAMs^2^. In species exhibiting a closed RAM, cell files corresponding to each tissue can be traced back to their original stem cells, so called initials. In the *Arabidopsis thaliana* closed RAM, slowly dividing cells corresponding to the quiescent center (QC) are surrounded by mitotically active initial cells forming the stem cell niche^3^. Initial cells generate transit-amplifying cells that divide several times within the RAM proliferation zone (PZ), start to differentiate in each root cell type at the transition zone (TZ), and rapidly expand in the elongation zone (EZ)^4,5^. In the model legume *Medicago truncatula*, the RAM shows a characteristic basic-open organization where initial cells are not clearly arranged in tiers around a limited number of QC cells^6^.

The RAM activity relies on a tight balance between cell division and differentiation, which notably results from interactions between auxin and cytokinin (CK) phytohormones^7,8^. In the *A*. *thaliana* RAM, CKs are proposed to promote cell differentiation and to restrict cell proliferation by directly activating the expression of the auxin repressor SHY2 (IAA3/SHORT HYPOCOTYL 2) depending on the CK signaling transcription factor ARR1 (ARABIDOPSIS RESPONSE REGULATOR 1)^9–11^. The resulting decrease of polar auxin transport and the limitation of auxin amounts supplied to the RAM may then restrict cell proliferation^9,12^. Another phytohormone, gibberellin (GA), was shown to interact with auxin and CK pathways in the RAM^10^. GAs are tetracyclic diterpenoid compounds, perceived in *A*. *thaliana* by GID1 (GIBBERELLIN INSENSITIVE DWARF 1), a soluble receptor which can interact with DELLA proteins and mediate their degradation by the 26S proteasome^13^. DELLAs belong to the GRAS nuclear protein family (GAI (GIBBERELLIC-ACID INSENSITIVE)/RGA (REPRESSOR OF GA1)/SCARECROW (SCR)) and act as central transcriptional repressors of GA responses. In *A*. *thaliana*, DELLA-mediated GA signaling negatively regulates root length by controlling the rate of meristematic cell division and elongation in the RAM^14,15^. The GA-insensitive gain of function *gai* mutant displays a delayed root growth while the quadruple-DELLA mutant *gai-t6 rga-t2 rgl1-1 rgl2-1* shows an increased cell proliferation. A targeted expression of a GA-insensitive *gai* allele specifically in the root endodermis also negatively affects cell elongation in the EZ^15^. In addition, it was observed that the distribution of the DELLA protein RGA is anticorrelated with the expression of a GA biosensor in the *Arabidopsis* RAM, and that cell length correlates with this GA gradient^16^. At the molecular level, it was proposed that high amounts of GAs in the *A*. *thaliana* TZ of the RAM induce the degradation of the DELLA protein RGA, resulting in the inactivation and decrease of the expression of the CK signaling transcription factor *ARR1*. This decrease, leading to the inhibition of the regulation of cell differentiation by CKs, then promotes auxin-dependent cell divisions^9,10,12^. In turn, auxin-dependent cell divisions reinforce GA biosynthesis through a positive feedback^17^.

In addition to root growth, hormonal crosstalks also regulate lateral root (LR) formation^18,19^. In *A*. *thaliana*, LR formation involves stereotyped cell divisions initiating from pericycle founder cells adjacent to protoxylem poles, resulting in the development of a primordium^20^. Auxins are required at different stages of LR development^21^ whereas CKs negatively regulate LR initiation^22–24^. Depending on plant species, different roles of GAs in LR formation have been reported. In *A*. *thaliana*, a GA-deficient *ga3ox1*/*ga3ox2* double mutant exhibits a decreased number of LRs which can be rescued by exogenous applications of GAs^25^. In contrast, in tomato and pepper, exogenous applications of GA-biosynthesis inhibitors stimulate LR formation^26,27^. Moreover, exogenous applications of GAs negatively regulate LR initiation in poplar, and transgenic GA-deficient (*35S:GA*_*2*_*ox1*) or GA-insensitive (*35S*:*rgl1*) lines both exhibit an increased number of LRs^28^.

In this study, we investigated the role of GAs and DELLA proteins in root development in the model legume *M*. *truncatula*, considering that its RAM shows a characteristic basic-open organization different from the Arabidopsis reference dicot plant. We found that GAs negatively regulate lateral root formation, as well as root growth depending on MtDELLA1. By using confocal microscopy, we showed that GAs primarily affect cell expansion in the RAM, not only longitudinally but also radially. This effect of GAs on cell elongation correlates with an inhibition of the transcriptional regulation of a subset of CK-induced expansin encoding genes, shown to be required for cell elongation in Arabidopsis^29^. In addition, GAs negatively regulate the number of cortical cell layers initiated in the apical part of the RAM, and consequently impact on the global root diameter.

## Results

### Gibberellins negatively regulate *M*. *truncatula* root growth depending at least on MtDELLA1

**Figure 1:**
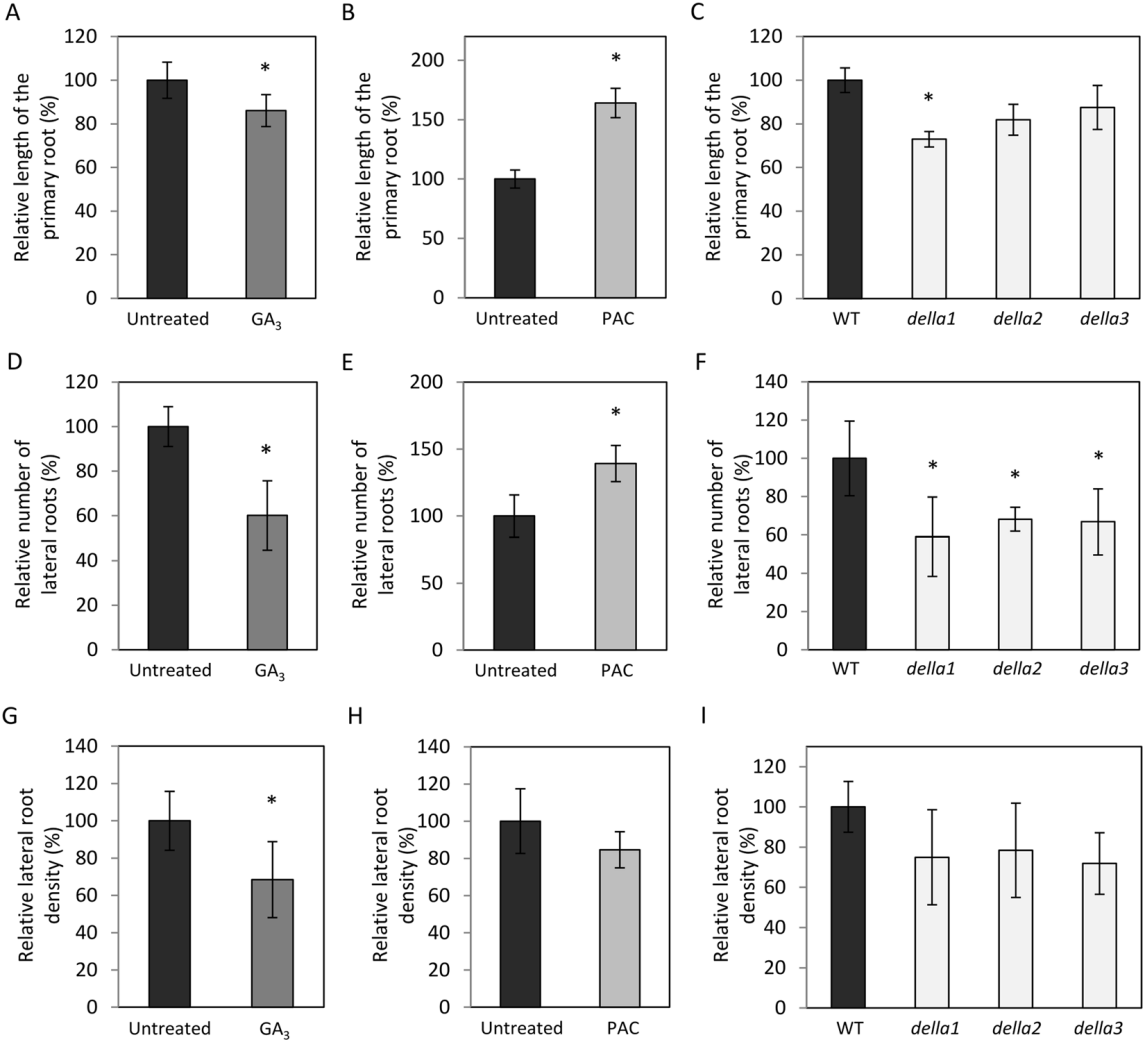
Gibberellins regulate root growth depending on MtDELLA1. (**A**) Relative length of wild-type (WT) untreated and GA_3_-treated primary roots. (**B**) Relative length of WT untreated and paclobutrazol (PAC)-treated primary roots. (**C**) Relative length of the primary root of WT untreated plants and of the *della1*, *della2* and *della3* mutants. (**D**) Relative number of lateral roots in WT untreated and GA_3_-treated plants. (**E**) Relative number of lateral roots in WT untreated and PAC-treated plants. (**F**) Relative number of lateral roots in WT untreated plants and in the *della1*, *della2* and *della3* mutants. (**G)** Relative lateral root density (number of lateral roots/cm of primary root) in WT untreated and GA_3_-treated plants. (**H**) Relative lateral root density in WT untreated and PAC-treated plants. (**I**) Relative lateral root density in WT untreated plants and in the *della1*, *della2* and *della3* mutants. In all cases, measurements were made two weeks post-germination. Error bars represent confidence interval (α = 0.05, n > 13 plants) of one representative biological experiment out of three, and asterisks indicate significant differences between the untreated control and treated samples, or WT and mutants, based on a Mann-Whitney test (α = 0.05).

To investigate the role of GAs in *M*. *truncatula* root development, we first characterized the effect of exogenous applications of bioactive GA_3_ and of the GA-biosynthesis inhibitor paclobutrazol (PAC) on the primary root growth of wild-type (WT) plants, two weeks post-germination (Fig. 1A,B). GA-treated plants displayed a shorter primary root compared to untreated plants (Fig. 1A) while a PAC treatment increased root length (Fig. 1B). These results thus point to a negative role of GAs in the regulation of *M*. *truncatula* primary root growth.

As DELLA proteins are critical regulators of GA-dependent responses^13^, and as previous studies conducted in *M*. *truncatula* reported that the three *MtDELLA* genes identified in the genome are expressed in the RAM^30–32^, we hypothesized that GAs could regulate primary root growth depending on DELLA proteins. We monitored the primary root growth of the three *della* mutants two weeks post-germination (Fig. 1C). Interestingly, the primary root length of the *della* mutants was reduced compared to WT plants, significantly for *della1*, suggesting that the inhibitory effect of GAs on primary root growth is mediated at least by MtDELLA1.

To evaluate the role of GAs and DELLA proteins in LR formation, we additionally assessed the effect of exogenous applications of GA_3_ and PAC on the number and density of emerged LRs in WT plants (corresponding to the number of LRs per cm of primary root; Fig. 1D,E,G,H). Both the number and density of LRs were decreased by a GA_3_ treatment compared to the untreated control (Fig. 1D,G). Conversely, a PAC treatment led to an increased number of emerged LRs (Fig. 1E) but had no significant effect on LR density (Fig. 1H). To evaluate the involvement of DELLA-dependent GA signaling in LR formation, we additionally quantified the number and density of emerged LRs in the three *della* mutants (Fig. 1F,I). While the number of LRs was decreased in all the *della* mutants (Fig. 1F), LR density did not significantly differ from the one of the WT (Fig. 1I). Altogether, these results indicate that the relationship between DELLA proteins and LR formation may indirectly, or at least partially, rely on the primary root growth phenotype. This suggests that a main feature of the DELLA-mediated GA signaling in *M*. *truncatula* roots is to positively regulate primary root growth.

### Gibberellins negatively regulate RAM size and the longitudinal expansion of cortical cells in *M*. *truncatula*

**Figure 2:**
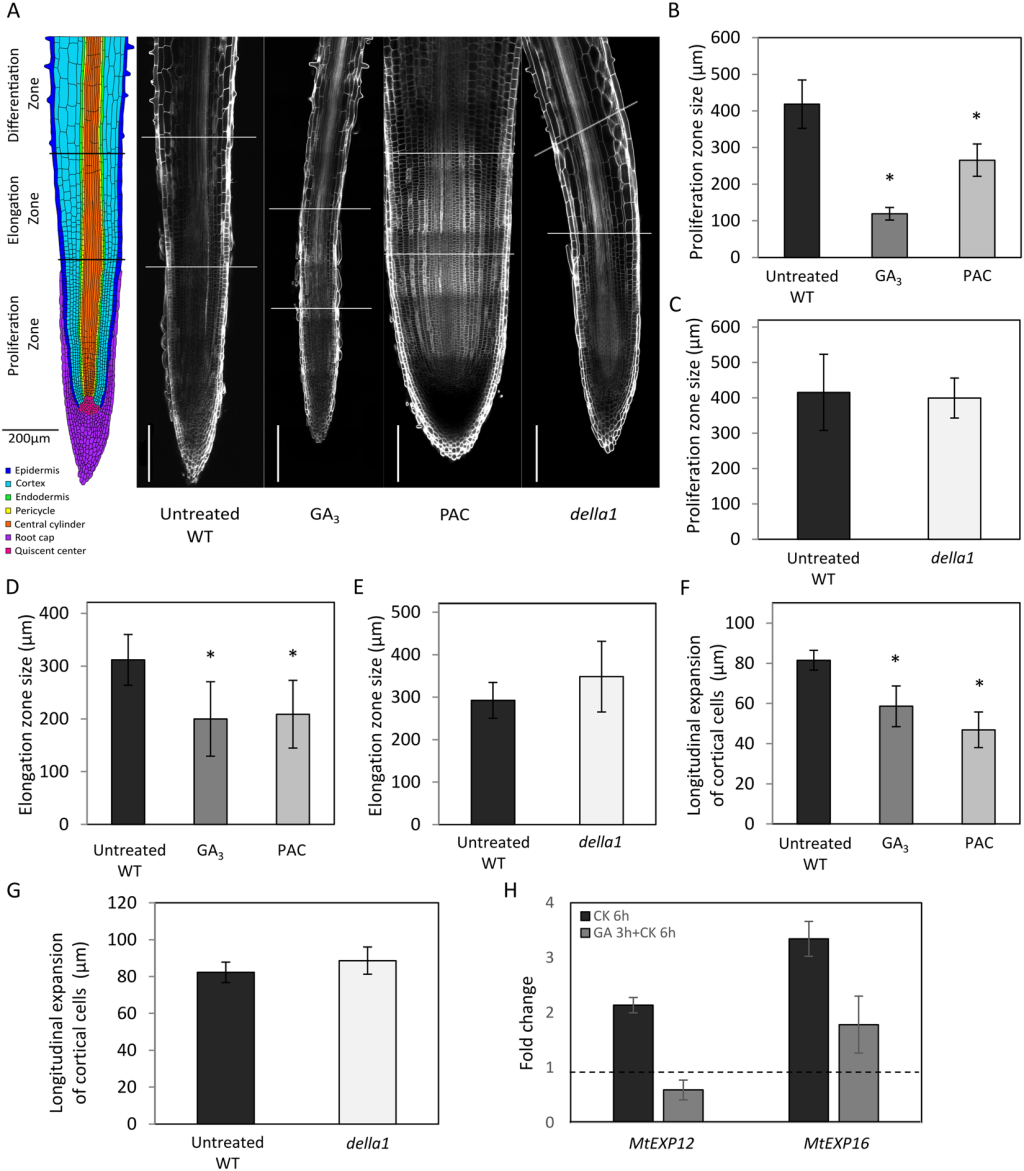
Gibberellins negatively regulate RAM size and the longitudinal expansion of cortical cells. (**A**) Scheme of a *Medicago truncatula* root and representative examples of wild-type (WT) untreated, GA_3_-treated, paclobutrazol (PAC)-treated, and *della1* mutant root apices cleared and counterstained with Renaissance to visualize cell walls. The proliferation (PZ) and elongation (EZ) zones are indicated with white lines. Bars = 200 µm. (**B**,**C**) Quantification of the PZ size in WT untreated, GA_3_-treated, PAC-treated roots (**B**), and in the *della1* mutant (**C**). (**D**,**E**) Quantification of the EZ size in WT untreated, GA_3_-treated, PAC-treated (**D**), and in the *della1* mutant (**E**). (**F**,**G**) Quantification of the longitudinal expansion of cortical cells in the EZ in WT untreated, GA_3_-treated, PAC-treated (**F**), and in the *della1* mutant (**G**). (**H**) Relative expression of *MtEXP12* and *MtEXP16* in WT roots after a CK (BAP) treatment preceded or not by a GA_3_ treatment. Transcript levels are normalized relative to the *MtACTIN11* reference gene and calibrated relative to mock-treated roots (the dotted line indicating a ratio of 1). In (**B**–**G**), measurements were made two weeks post-germination. Error bars represent confidence interval (α = 0.05; n > 5 plants) of one representative biological experiment out of two, and asterisks indicate significant differences between the untreated control and treated samples, or WT and mutants, based on a Mann-Whitney test (α = 0.05). In (**H**), error bars represent standard deviation.

To explain the negative regulation of primary root growth by GAs, we hypothesized that MtDELLA1-mediated GA signaling could modulate PZ and EZ length in the RAM. To visualize cells within the whole *M*. *truncatula* RAM without performing any section, a clearing protocol was developed followed by a “Renaissance” staining of cell walls (Fig. 2A)^33^. A significant reduction of both PZ (Fig. 2B) and EZ size (Fig. 2D) was detected in the RAM of GA-treated plants but not in the *della1* mutant (Fig. 2C,E) previously showing a significant reduction of root length (Fig. 1C). Unexpectedly, the size of the PZ and EZ was also significantly decreased in response to PAC (Fig. 2B,D). Additionally, the quantification of the maximal longitudinal elongation of cortical cells at the basal end of the EZ suggests that the reduction of the EZ length in response to both GAs and PAC correlates with a reduced longitudinal expansion of cortical cells (Fig. 2F). No significant change in the longitudinal expansion of cortical cells was detected in the *della1* mutant (Fig. 2G). Taken together, these data indicate that GAs may regulate root growth by negatively controlling EZ and PZ size, as well as the longitudinal expansion of cortical cells.

In the Arabidopsis distal meristem, changes in cell elongation have been associated with the activation of a subset of expansin (EXPA) proteins whose expression is induced by CKs^29^. We therefore identified in *M*. *truncatula* the proteins most closely related to the Arabidopsis CK-activated AtEXPA1, AtEXPA10, AtEXPA14, and AtEXPA15, recently linked to the regulation of RAM size^29^. Two proteins were identified, previously named MtEXPA12 and MtEXPA16^34^. The expression of *MtEXPA12* and *MtEXPA16* is induced in *M*. *truncatula* roots by a short-term CK treatment (BAP 10^−7^ M for 6 h; Fig. 2H), suggesting the conservation of this regulation with their Arabidopsis homologs^29^. Interestingly, a GA_3_ pre-treatment (10^−6^ M for 3 h) suppressed the CK-induction of *MtEXPA12* and highly reduced the CK-induction of *MtEXPA16* (Fig. 2H). These results thus suggest that in *M*. *truncatula*, GAs may negatively regulate cortical cell elongation, and consequently EZ and PZ size, through the regulation of CK-induced *EXPA* genes.

### Gibberellins negatively regulate root diameter and radial cell expansion in the RAM of *M*. *truncatula*

**Figure 3:**
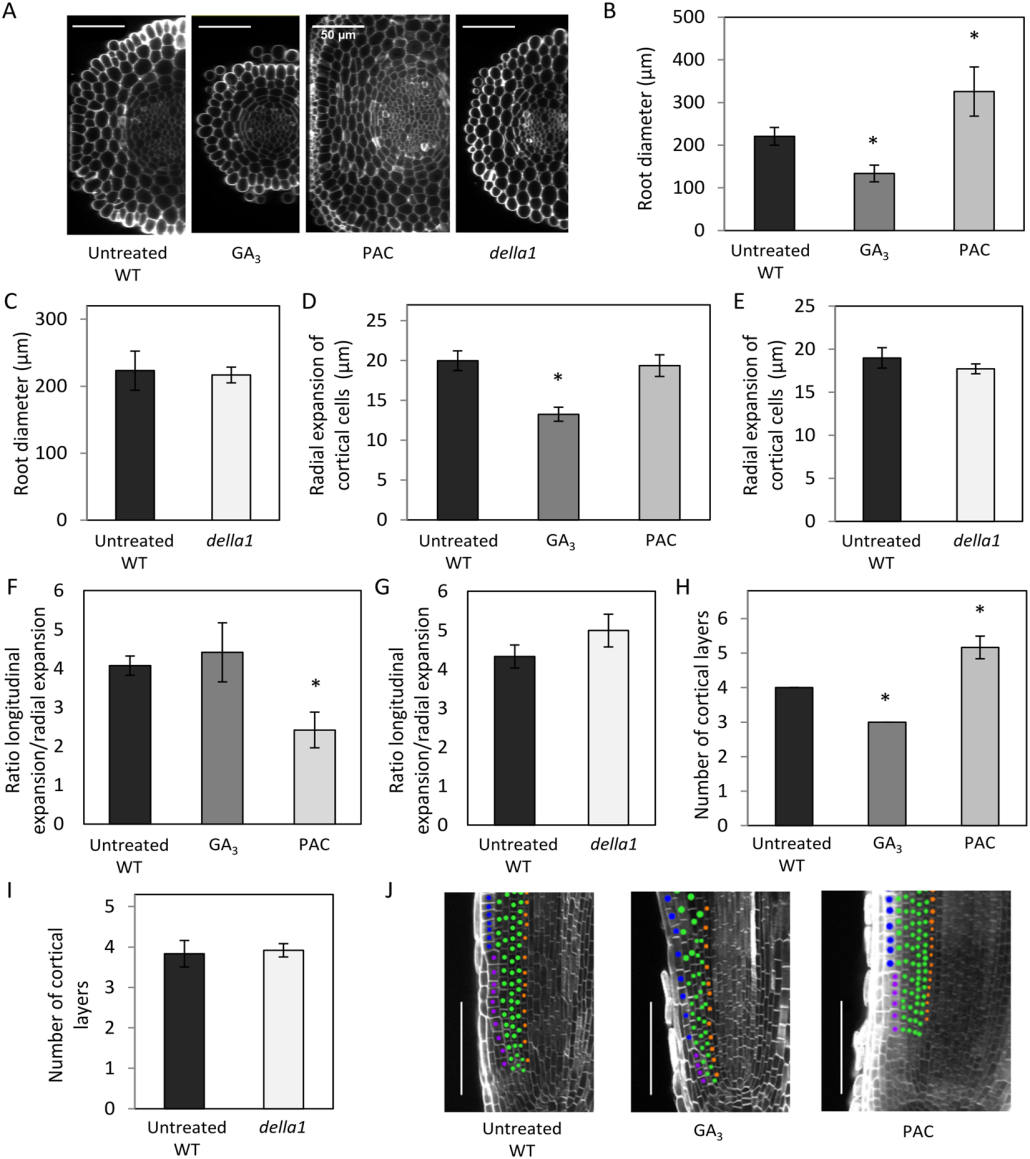
Gibberellins negatively regulate the radial expansion of cortical cells and the number of cortical cell layers. (**A**) Representative confocal optical transversal section of wild-type (WT) untreated, GA_3_-treated, paclobutrazol (PAC)-treated, and *della1* mutant root apices cleared and counterstained with Renaissance to visualize cell walls. Bars = 50 µm. (**B**,**C**) Quantification of the root diameter in WT untreated, GA_3_-treated, PAC-treated roots (**B**), and in the *della1* mutant (**C**). (**D**,**E**) Quantification of the radial expansion of outer cortical cells in the elongation zone (EZ), in WT untreated, GA_3_-treated, PAC-treated roots (**D**), and in the *della1* mutant (**E**). (**F**,**G**) Ratio between the longitudinal expansion and the radial expansion of outer cortical cells in the EZ in WT untreated, GA_3_-treated, PAC-treated roots (**F**), and in the *della1* mutant (**G**). (**H**,**I**) Quantification of the number of cortical layers in WT untreated, GA_3_-treated, PAC-treated roots (**H**), and in the *della1* mutant (**I**). (**J**) Representative example of the stem cell niche organization in WT untreated, GA_3_-treated, and PAC-treated root apices. Colored dots highlight the different cell files from the outside to the inside: epidermis (blue), cortex (green), and endodermis (orange). Bars = 100 µm. In (**B**–**I**), measurements were made two weeks post-germination. Error bars represent confidence interval (α = 0.05; n > 5 plants) of one representative biological experiment out of two, and asterisks indicate significant differences between the untreated control and treated samples, or WT and mutants, based on a Mann-Whitney test (α = 0.05).

As GAs affected the longitudinal elongation of cortical cells in the RAM, we wondered if the radial expansion and global root diameter were also impacted at the root apex. We therefore measured the root diameter of GA- or PAC-treated plants, and of the *della1*, *della2* and *della3* single mutants (Fig. 3A–C; Supplementary Fig. 1A,B). An exogenous GA_3_ treatment significantly decreased the root diameter compared to the WT (Fig. 3B; Supplementary Fig. 1B), whereas no change was detected in *della* mutants, which were still responsive to GA (Fig. 3C; Supplementary Fig. 1A,B). Conversely, a PAC treatment significantly increased the root diameter (Fig. 3B). To determine if this effect was related to cell expansion on the radial axis, we then measured cell expansion of cortical cells on RAM optical transversal sections taken at the basal end of the EZ (Fig. 3A,D,E; Supplementary Fig. 1A,D). As previously observed for longitudinal cell expansion (Fig. 2F), GA_3_ reduced the radial expansion of cortical cells (Fig. 3D), while PAC-treated roots did not show any modification of radial cell expansion (Fig. 3D). No significant difference in the radial expansion of cortical cells was detected in *della* mutant roots, which were still sensitive to GA (Fig. 3E; Supplementary Fig. 1A,D). Consequently, the typical longitudinal/radial expansion ratio of cortical cells was lost in PAC-treated roots compared to untreated WT, GA-treated, or *della* mutant roots, leading to the formation of cells with a more squared shape (Figs. 3A,F,G; Supplementary Fig. 1F). This result suggests that GAs regulate cortical cell elongation in both longitudinal and radial axes in the *M*. *truncatula* RAM.

Interestingly, these changes in root diameter were additionally correlated to modifications of the number of root cortical cell layers in the RAM, whereas epidermal, endodermal and pericycle cell layers, identified based on their specific features (respectively root hair formation, cell shape, and cell size) were retrieved (Fig. 3H,I; Supplementary Fig. 1A,C). Indeed, GA-treated roots displayed less cortex layers, while a PAC-treatment increased the number of cortex layers (Fig. 3H; Supplementary Fig. 1C). No additional or missing cortex layer was however detected in the three *della* mutants, which were still responsive to GA (Fig. 3I; Supplementary Fig. 1A,C). To determine the origin of the additional cortical layers formed in PAC-treated roots, we analyzed more closely the *M*. *truncatula* RAM (Fig. 3J). Even though the cell layers could not be followed until a very tightly localized QC/initial region such as the one described in the *A*. *thaliana* closed RAM, we showed that the additional cell file in PAC-treated roots and the missing cell file in GA-treated roots could be traced back in the most apical region of the RAM (Fig. 3J). Overall, these results suggest that GAs decrease root diameter by affecting both radial expansion of cortical cells and the number of cortical layers that form at the PZ apex.

## Discussion

Root system architecture is continuously regulated by environmental cues. This developmental plasticity, enabling plants to overcome constraints due to their sessile nature, is intrinsically controlled by phytohormones. In addition to the well-described auxin and CK pathways^35^, several studies conducted in different plant species highlighted contrasting roles for GA signaling in root system development. Our work revealed that in contrast to Arabidopsis^14,15,25^, GA-treated plants and the three single *della* mutants displayed a reduction of both primary root growth and LR number at two weeks post-germination, illustrating that diverse involvements of GA signaling in the regulation of root system development exist. Even though GAs and *della* mutations negatively affected root growth and LR formation, the LR density of the three *della* mutants did not significantly differ from the one of the WT. This suggests that the main feature of DELLA-mediated GA signaling in the regulation of *M*. *truncatula* root system architecture may be to negatively regulate root growth. In addition, the fact that the *della1* mutant exhibited a stronger root phenotype than *della2* and *della3* mutants suggest that the regulation of root growth by GAs may be mostly mediated by MtDELLA1. However, while the root length of the *della1* mutant was reduced, no significant difference in PZ and EZ size was detected in the RAM, suggesting that this macroscopic effect cannot be resolved into discrete microscopic effects.

In *A*. *thaliana*, root growth was proposed to be regulated by GAs from the endodermis^14,15^. Indeed, the disruption of GA responses through the expression of a dominant active DELLA *gai* specifically in the endodermis was sufficient to reduce endodermal cell elongation and RAM size^15^, and consistently a preferential accumulation of exogenous fluorescein-GA_3_ and fluorescein-GA_4_ in endodermal cells of the EZ was observed by Shani *et al*.^36^. However, it was recently shown by using a GA biosensor that GAs also accumulate in epidermal and cortical cells of the EZ, suggesting that the action of GAs is not limited to the root endodermis^16^. In *M*. *truncatula*, we observed that GAs negatively affected longitudinal and radial expansion of cortical cells, further suggesting that the action of GAs may not be restricted to the endodermis.

In Arabidopsis, cell wall expansion in the distal RAM was recently shown to rely on a subset of CK-induced expansin genes, allowing to regulate the position of the cell differentiation/proliferation transition zone, and consequently RAM size and root growth^29,37,38^. Accordingly, we identified in *M*. *truncatula* two homologs of these expansins, *MtEXPA12* and *MtEXPA16*, whose expression is rapidly induced by CKs in roots. Interestingly, this CK-induction was decreased by a GA pre-treatment, suggesting that GAs may negatively regulate cell elongation in *M*. *truncatula* roots through the inhibition of CK-induced expansins that control RAM size in Arabidopsis.

Additionally, we noticed that GA- and PAC-treated roots respectively displayed a decreased and increased diameter, in part due to missing or additional cell files in the apical region of the RAM. In Arabidopsis and rice, a related effect of GAs and PAC on the number of cell files and root diameter was also reported^39,40^. As Arabidopsis RAM only contains a single layer of cortex, it was proposed that GAs delayed the onset of the formation of the middle cortex while PAC induced its precocious formation^39^. At the molecular level, it was proposed both in Arabidopsis and rice that the ectopic expression of the mobile SHORT-ROOT transcription factor increases the number of cortex layers^41,42^. More recently, it was shown that the formation of two layers of cortex in *Cardamina hisrsuta*, a close relative to the single cortex-layered *A*. *thaliana* reference plant, relies on the activity of miR165/166 microRNAs regulating HD-ZIP III transcription factors required for the formation of the inner cortex layer^41,42^. Here, we showed in *M*. *truncatula* that missing cell files in GA-treated roots and additional cell files in PAC-treated roots could be traced back in the most apical part of the RAM. Even though the lack of available markers did not allow us to definitely conclude on the nature of the affected cell file, typical cell features of epidermal, endodermal and pericycle cell layers, respectively corresponding to root hair differentiation, cell shape, and cell size, could be identified in GA-treated roots. This strongly suggests that it is the number of cortical cell layers that is affected, in agreement with data gained in Arabidopsis^37,39^ and in the region of *M*. *truncatula* roots responding to symbionts^43^.

The altered root diameter of GA- and PAC-treated roots is additionally linked to a change in the radial width of RAM cortical cells, consistently with observations from Heck *et al*.^43^ in the symbiotic susceptible zone. Whereas GAs and PAC unexpectedly both decreased longitudinal expansion of cortical cells, only GAs additionally reduced radial cell expansion. This suggests a differential effect of the PAC treatment depending on the orientation of cortical cell expansion, consequently leading to the formation of cells having lost their typical rectangular shape towards a squarer shape. Further analyses would be required to determine which targets of PAC action may explain its differential effect on radial *versus* longitudinal cortical cell expansion in the *M*. *truncatula* RAM. The three *della* mutants did not reveal any significant root diameter phenotype, even though in some *della1* and *della2* mutant roots the number of cortical cell layers was decreased (Supplementary Fig. 1C), suggesting that DELLA1 and DELLA2 participate in regulating root diameter and cortex patterning. As each *della* mutant is still responsive to GA for most of the parameters analyzed, this also strongly suggests that a functional redundancy exists between DELLA proteins which have a largely overlapping expression pattern in the RAM^31^. Accordingly, the use of *della1-della2* double mutants revealed a decreased radial cell expansion of cortical cells in the symbiotic responsive zone located just above the RAM^43^. Finally, similar to the Arabidopsis precocious induction of the middle cortex^39^, we observed related asynchronous periclinal divisions of individual cells at the apex of the inner cortex layer, with a coexistence of divided and undivided cells interspaced in the same cell file. As in *M*. *truncatula* the additional or missing cell file is detected very close to RAM initial cells, this suggests that GAs may be involved in the set-up of the root multicortex pattern.

In legumes, the regulation of cortical cell expansion and divisions in the region responsive to symbiotic microbes was proposed to be critical for the establishment of endosymbioses^43,44^. Indeed, while the development of endomycorrhizal arbuscules requires the expansion of cortical cells^40,43^, symbiotic nodulation involves a root-derived lateral organogenesis initiated by the activation of cortical cell divisions^44^. This suggests that in addition to direct functions of GAs and DELLA proteins on the infection by fungi or rhizobial bacteria^30–32,45^, the function of GAs in the regulation of cortical cell expansion and patterning (i.e. the number of cell files) could indirectly influence the root ability to establish symbiotic interactions^38^. GAs and DELLA proteins were previously proposed to regulate root nodule organogenesis^45–47^, with positive or negative roles reported depending on plant species and approaches used, but such pleiotropic effects of GAs on root cortex development within the symbiotic responsive zone make difficult to infer definitive conclusions about a precise function of GAs in symbiotic nodule organogenesis. Overall, GAs exert a complex regulation of cell expansion and patterning in the RAM, which might be different depending on plant species and likely indirectly affect symbiotic interactions and nodule root-derived organogenesis.

## Methods

### Plant material and treatments

Seeds of the *M*. *truncatula* genotype Jemalong R108 were used in this study. The *della1* (NF12399), *della2* (NF4302) and *della3* (NF10539) mutant lines are described in Fonouni-Farde *et al*.^31^. Seeds were scarified and sterilized as described in Gonzalez-Rizzo *et al*. (2006)^48^.

For *in vitro* pharmacological treatments, germinated R108 seeds were grown vertically on a growth paper (Mega International; http://www.megainternational.com/index.htm) on a “i” medium^49^ supplemented with 1.5% Bacto-Agar (Gibco), in growth chambers at 24 °C under long-day conditions (16 h light at 150μE light intensity/8 h dark). After two days, the growth paper carrying the plants was transferred on a fresh “i” medium with or without GA_3_ (0,1 µM, Sigma-Aldrich) or paclobutrazol (PAC, 0,01 µM, Sigma-Aldrich), defined as the minimal concentrations leading to significant effects on root development. Root length was measured using the ImageJ software and emerged LRs were quantified two weeks post-germination.

For hormonal treatments, seedlings grown on a grid in a Magenta box containing a liquid “i” medium were pre-treated for 3 h with 10^−6^ M GA_3_ (Sigma-Aldrich) and then treated for 6 h with 10^−7^ M BAP (Sigma-Aldrich). Control experiments performed in parallel consisted in mock-treated samples. Roots were collected and immediately frozen in liquid nitrogen.

### RAM clearing and staining

Root tips (2 cm long) were cut and directly cleared into a NaOH 0.8% – SDS 20% solution^50^. After 2 h of incubation at 37 °C, samples were rinsed two times with water, treated with 5% bleach for 30 min at room temperature, and again rinsed two times with water. Samples were then stained with a solution containing Renaissance 2200 (Renaissance chemicals Ltd, UK) as described in Musielak *et al*.^33^, and vacuum-infiltrated for 15 min. Samples were stored at 4 °C for one to three days, depending on their size, before observation.

### Image acquisition and image analysis

Samples were mounted between a slide and a coverslip with a handmade tape spacer adapted to the diameter of the root samples and visualized under a Zeiss LSM880 Laser Scanning Confocal Microscope. The Renaissance staining was visualized using a 405 nm laser line excitation and a 410–500 nm emission window. Images were acquired with a 40x objective (numerical aperture: 1,2) using a Z-scan appropriate to cover the whole root width at the end of the EZ zone, easily tractable by the bulging of root hairs^51^. Stitching was used to assemble mosaic images allowing covering the whole RAM. Images were then analyzed using Image J, and different parameters were measured. The PZ zone was defined from the initials to the RAM region where cells of the outer cortex layer showed a double longitudinal size as the average PZ cells. The EZ was defined from the PZ basal end towards the first bulging root hair corresponding to the end of outer cortex cell elongation^51^. Cell longitudinal expansion was measured at the end of the EZ on the outer layer of cortical cells. Root diameter and cell radial expansion were measured on optical transversal sections generated at the end of the EZ. To mark the different cell files in the RAM, the iRoCS toolbox was used (intrinsic Root Coordinate System, http://www.plant-image-analysis.org/software/irocs-toolbox)^52^.

### Gene Expression Analysis

RNA extraction, cDNA synthesis, and real-time RT-PCR experiments were performed as described by Fonouni-Farde *et al*.^31^, using primer combinations showing a minimum amplification efficiency of 90% (Supplementary Table 1). Reference genes used were *MtACTIN11* and *MtRBP1* (RNA Binding protein 1), previously validated using the Genorm software in these conditions. Two independent biological experiments were performed, with two technical replicates for each condition.

### Statistical analyses

Statistical analyses were performed with a non-parametric Mann-Whitney test to compare the effect of treatments relatively to the non-treated control, and mutants relatively to the WT control; or with a Kruskal-Wallis test when all genotypes and treatments were compared.

## Supplementary information

Supplementary Figure 1 and Table 1

## Data Availability

Datasets generated or analysed during this study are included in this published article (and its Supplementary Information files).
