# Supplementary material for: Gibberellins negatively regulate the development of Medicago truncatula root system
Source: Sci Rep. 2019 Feb 20;9:2335. doi: 10.1038/s41598-019-38876-1 (PMC6382856; doi:10.1038/s41598-019-38876-1)
Supplement: Supplementary file 1 — Supplementary Figure 1 and Table 1 [file 41598_2019_38876_MOESM1_ESM.pdf]

Gibberellins negatively regulate the development of *Medicago truncatula* root system

Camille Fonouni-Farde, Ambre Miassod, Carole Laffont, Halima Morin, Abdelhafid Bendahmane, Anouck Diet and Florian Frugier

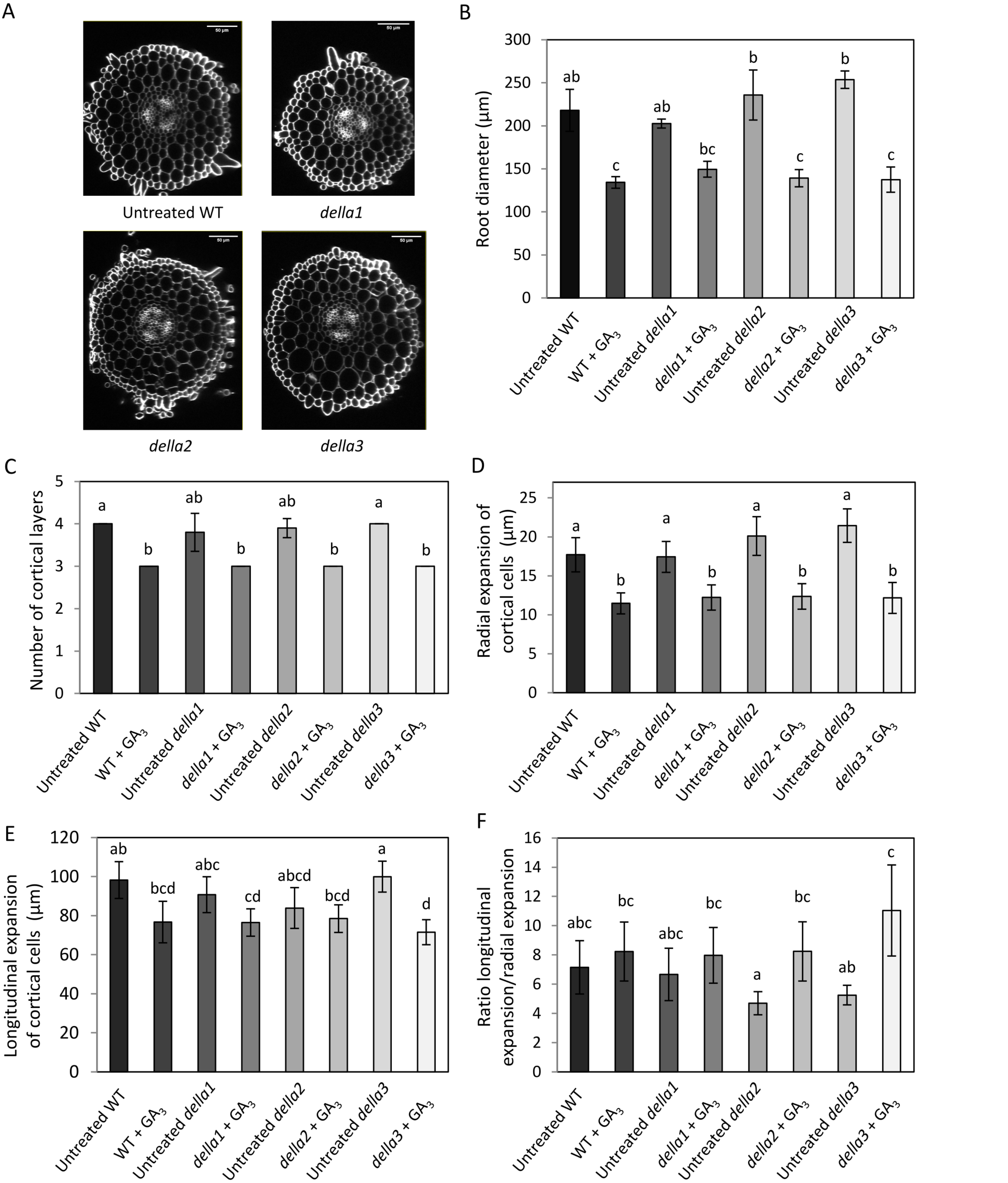

Supplementary Figure 1. Gibberellins negatively regulate the root diameter, the expansion of cortical cells, and the number of cell files in *della* mutants.

(A) Representative confocal optical transversal section of wild-type (WT) untreated, *della1*, *della2*, and *della3* mutants root apices cleared and counterstained with Renaissance to visualize cell walls. Bars=50 µm. (B) Quantification of the root diameter in WT, *della1*, *della2*, and *della3* mutant roots treated or not with GA_3. (C) Quantification of the number of cortical layers in WT, *della1*, *della2*, and *della3* mutant roots treated or not with GA_3. (D) Quantification of the radial expansion of outer cortical cells in the elongation zone (EZ), in WT, *della1*, *della2*, and *della3* mutant roots treated or not with GA_3. (E) Quantification of the longitudinal expansion of outer cortical cells in the elongation zone (EZ), in WT, *della1*, *della2*, and *della3* mutant roots treated or not with GA_3. (F) Ratio between the longitudinal expansion and the radial expansion of outer cortical cells in the EZ in WT, *della1*, *della2*, and *della3* mutant roots treated or not with GA3.

In (B-F), measurements were made two weeks post-germination. Error bars represent confidence interval (α=0.05; n>5 plants) and letters indicate significant differences between all genotypes and treatments based on a Kruskal-Wallis test (α=0.05).

**Gene name**	**Gene ID (Mtv4.0)** (available at https://phytozome.jgi.doe.gov/)	**Forward primer (5' to 3')**	**Reverse primer (5' to 3')**
MtACTIN	Medtr7g026230	TGGCATCACTCAGTACCTTTCAACAG	ACCCAAAGCATCAAATAATAAGTCAACC
MtRBP1	Medtr6g034835	AGGGGCAAGTTCCTTCATTT	AAACGGACGGAAAATGTGAG
MtEXPA12	Medtr4g081950	AGGCCAATGAAGGGCATATT	TAGCGGGTGGTATCCATTTC
MtEXPA16	Medtr5g041700	TAGGTGTGCTGGTGACCGTA	GAGGGTTACACCATCCTCCA
